# Effects of Laser Printer–Emitted Engineered Nanoparticles on Cytotoxicity, Chemokine Expression, Reactive Oxygen Species, DNA Methylation, and DNA Damage: A Comprehensive *in Vitro* Analysis in Human Small Airway Epithelial Cells, Macrophages, and Lymphoblasts

**DOI:** 10.1289/ehp.1409582

**Published:** 2015-06-16

**Authors:** Sandra V. Pirela, Isabelle R. Miousse, Xiaoyan Lu, Vincent Castranova, Treye Thomas, Yong Qian, Dhimiter Bello, Lester Kobzik, Igor Koturbash, Philip Demokritou

**Affiliations:** 1 Department of Environmental Health, Center for Nanotechnology and Nanotoxicology, Harvard T.H. Chan School of Public Health, Harvard University, Boston, Massachusetts, USA; 2 Department of Environmental and Occupational Health, University of Arkansas for Medical Sciences, Little Rock, Arkansas, USA; 3 Department of Pharmaceutical Sciences, West Virginia University, Morgantown, West Virginia, USA; 4 Office of Hazard Identification and Reduction, U.S. Consumer Product Safety Commission, Rockville, Maryland, USA; 5 Pathology and Physiology Research Branch, Health Effects Laboratory Division, National Institute for Occupational Safety and Health, Morgantown, West Virginia, USA; 6 Department of Work Environment, University of Massachusetts-Lowell, Lowell, Massachusetts, USA

## Abstract

**Background:**

Engineered nanomaterials (ENMs) incorporated into toner formulations of printing equipment become airborne during consumer use. Although information on the complex physicochemical and toxicological properties of both toner powders and printer-emitted particles (PEPs) continues to grow, most toxicological studies have not used the actual PEPs but rather have primarily used raw toner powders, which are not representative of current exposures experienced at the consumer level during printing.

**Objectives:**

We assessed the biological responses of a panel of human cell lines to PEPs.

**Methods:**

Three physiologically relevant cell lines—small airway epithelial cells (SAECs), macrophages (THP-1 cells), and lymphoblasts (TK6 cells)—were exposed to PEPs at a wide range of doses (0.5–100 μg/mL) corresponding to human inhalation exposure durations at the consumer level of 8 hr or more. Following treatment, toxicological parameters reflecting distinct mechanisms were evaluated.

**Results:**

PEPs caused significant membrane integrity damage, an increase in reactive oxygen species (ROS) production, and an increase in pro-inflammatory cytokine release in different cell lines at doses equivalent to exposure durations from 7.8 to 1,500 hr. Furthermore, there were differences in methylation patterns that, although not statistically significant, demonstrate the potential effects of PEPs on the overall epigenome following exposure.

**Conclusions:**

The *in vitro* findings obtained in this study suggest that laser printer–emitted engineered nanoparticles may be deleterious to lung cells and provide preliminary evidence of epigenetic modifications that might translate to pulmonary disorders.

**Citation:**

Pirela SV, Miousse IR, Lu X, Castranova V, Thomas T, Qian Y, Bello D, Kobzik L, Koturbash I, Demokritou P. 2016. Effects of laser printer–emitted engineered nanoparticles on cytotoxicity, chemokine expression, reactive oxygen species, DNA methylation, and DNA damage: a comprehensive *in vitro* analysis in human small airway epithelial cells, macrophages, and lymphoblasts. Environ Health Perspect 124:210–219; http://dx.doi.org/10.1289/ehp.1409582

## Introduction

The recent incorporation of engineered nanomaterials (ENMs) into toner formulations has potential health implications based on consumer exposure to released particulate matter (PM) from laser-based printing equipment. Laser printers are widely used in office and home environments, and there has been an exponential increase of market sales in recent years (IDC 2014). Recent studies have shown that emissions from this growing technology comprise a variety of pollutants including PM, semi-volatile organic compounds (sVOCs), and other gaseous pollutants (He et al. 2007; Morawska et al. 2009; Wang et al. 2012).

Recently, our group developed a laboratory-based printer exposure generation system (PEGS) that allows generation and sampling of airborne printer-emitted particles (PEPs) for subsequent physicochemical, morphological, and toxicological analysis (Pirela et al. 2014). This platform was used to evaluate emission profiles from 11 laser printers that are currently on the market. The study showed that the particle concentration of PEPs varied across printers/manufacturers, with printers emitting as much as 1.3 million particles/cm^3^ with diameters < 200 nm (Pirela et al. 2014). The detailed assessment of both toners and PEPs confirmed the presence of nanoscale materials in the airborne state and revealed the complex chemistry of these materials, which included elemental/organic carbon and inorganic compounds (e.g., metals, metal oxides). These findings confirmed that toners are nanoenabled products (NEPs) (Pirela et al. 2015).

Both *in vitro* and *in vivo* toxicological assays may help characterize the effects of laser printer emissions and toners on the respiratory system. However, the results obtained to date are contradictory. Notably, the toxicity of PEPs remains poorly characterized primarily because most studies have used toner powders rather than PEPs. For example, Gminski et al. (2011) reported that toner powders exhibited genotoxic potential on epithelial lung cells. Similar *in vitro* assays using an air/liquid interphase system showed significant cyto- and genotoxicity (Tang et al. 2012). In contrast, cell magnetometry analysis of alveolar macrophages exposed to toner powder revealed no effects (Furukawa et al. 2002). An even smaller number of *in vivo* toxicological studies have evaluated the effects of exposure to PEPs. Bai et al. (2010) reported that mice exposed to printer toner particles showed significant pulmonary inflammation, damage to the epithelial–capillary barrier, and enhanced cell permeability. Comparable inflammatory and fibrotic responses were also observed in rats exposed to toner powders (Morimoto et al. 2013).

Concerns continue to be raised with regard to the possible epigenetic effects associated with PEP inhalation exposure. In general, the ability of ENMs to affect the cellular epigenome remains largely unexplored. One important epigenetic mechanism, DNA methylation, can regulate the proper expression of genetic information in a sex-, tissue-, and cell type–dependent manner (Jones 2012). Additionally, DNA methylation plays a central role in regulating the expression of transposable elements (TEs) that comprise a large part of the eukaryotic genome (Smith et al. 2012). TEs are essential regulators of the stability and proper function of the genome, including the expression of genetic information and chromatin structure. Numerous studies indicate that exposure to various environmental stressors, including PM, may compromise the methylome and TEs (Baccarelli et al. 2009; Madrigano et al. 2011). An *in vitro* study by Gong et al. (2010) concluded that short-term exposure of human keratinocytes to nanomaterials might result in alterations of both global DNA methylation patterns and the DNA methylation machinery. However, the epigenetic effects of ENMs contained in PEPs remain largely unknown, and, to our knowledge, the use of *in vitro* systems to characterize epigenetic effects resulting from exposure to PEPs has not yet been done.

In the present *in vitro* toxicological study, the biological responses occurring upon exposure to a wide range of doses of PEPs were evaluated using physiologically relevant cells: human small airway epithelial cells (SAECs), macrophages (THP-1 cells), and lymphoblasts (TK6 cells). In this study, several endpoints important for understanding mechanisms of toxicity (e.g., cell membrane integrity, ROS production, DNA methylation) were assessed taking into consideration *in vitro* and *in vivo* dosimetry. Such thorough physicochemical, morphological, and cellular toxicological studies based on “real-world” exposure conditions add to the body of scientific evidence required to understand and quantify the risk of exposure to PEPs with the use of printing equipment. More importantly, the proposed methodology can be used to assess risks associated with ENMs released throughout the life cycle of any nanoenabled product.

## Materials and Methods

### Generation and Collection of Size-Fractionated PEPs

The PEPs were generated using the recently developed PEGS as described in our publication (Pirela et al. 2014). In summary, the PEGS was used to generate, collect, and sample size-fractionated PEPs from a high-emitting printer [referred to as Printer B1 in companion papers (Pirela et al. 2014, 2015)] that emitted up to 1.26 million particles/cm^3^ (Pirela et al. 2014, 2015).

### Postsampling Physicochemical and Morphological Characterization of PEPs

Detailed chemical and morphological characterization of the PEPs and toner from the test printer, as well as the paper utilized in the present study, are presented in detail in a recently published companion publication (Pirela et al. 2015). In summary, the toner powder and PEPs share a similar chemical fingerprint, containing 62% and 97% organic carbon, respectively; 10% and 0.5% elemental carbon, respectively; approximately 3% metal/metal oxides (e.g., aluminum, titanium); and approximately 25% other elements (e.g., phosphorus, sulfur) (Pirela et al. 2015).

### Extraction of Size-Fractionated PEPs and Preparation and Characterization of Particle Liquid Suspensions for Cellular Studies

After sampling size-fractionated PEPs, the particles were extracted from collection filter media using aqueous suspension methodology (Demokritou et al. 2002; Pirela et al. 2015). Subsequently, particle dispersions in culture media were prepared using a protocol developed by the authors (Cohen et al. 2013), in which the particle critical delivered sonication energy (DSE_cr_), hydrodynamic diameter (d_H_), formed agglomerate size distribution, polydispersity index (PdI), zeta potential (ζ), specific conductance (σ), pH, colloidal stability, and effective density of formed agglomerates (DeLoid et al. 2014) were measured. The PEP dispersion values are presented in Table 1. Before being used in experiments, the particle suspensions were prepared with sterile deionized water (DI H_2_O) and were sonicated at DSE_cr_, then diluted to the desired final test concentrations in media. It is noteworthy that the effective density of the formed agglomerates, which plays an important role in *in vitro* settling and dosimetry, was measured using the recently developed volumetric centrifugation method (VCM) (DeLoid et al. 2014).

**Table 1:** Properties of laser printer–emitted particle dispersions.

Material/media	d_H_ (nm)	PdI	ζ (mV)	σ (mS/cm)	ρ_agg_ (g/cm^3^)
PEPs (PM_0.1_)
DI H_2_O	178.3 ± 3.459	0.403 ± 0.050	–20.6 ± 1.87	0.185 ± 0.00058	—
RPMI/10% HS	272.5 ± 22.27	0.688 ± 0.178	–9.80 ± 1.31	3.61 ± 0.246	1.19
RPMI/10% FBS	227.3 ± 105.0	0.485 ± 0.247	9.55 ± 2.89	7.01 ± 0.960	1.56
SAGM	381.7 ± 40.23	0.586 ± 0.048	9.97 ± 2.77	2.52 ± 0.0721	2.39
Mild steel welding fumes (MS-WF)
DI H_2_O	2197 ± 118.4	0.561 ± 0.325	8.52 ± 1.24	0.028 ± 0.000093	—
RPMI/10% HS	1878.3 ± 395.89	0.236 ± 0.080	10.5 ± 0.757	11.9 ± 0.289	1.48
RPMI/10% FBS	1502 ± 96.26	0.236 ± 0.080	12.1 ± 2.66	11.5 ± 1.10	1.56
SAGM	1526.7 ± 259.63	0.198 ± 0.041	18.8 ± 0.9	10.5 ± 0.462	1.37
SiO_2_
DI H_2_O	142.5 ± 2.364	0.207 ± 0.013	33.6 ± 1.70	0.008 ± 0.000044	—
RPMI/10% HS	173.4 ± 13.36	0.541 ± 0.027	11.4 ± 3.60	11.2 ± 0.874	1.3
RPMI/10% FBS	114.6 ± 0.100	0.324 ± 0.009	9.33 ± 0.841	11.6 ± 0.833	1.2
SAGM	207.7 ± 6.029	0.583 ± 0.078	12.7 ± 1.39	11.1 ± 0.436	1.12
Abbreviations: —, data not available; d_H_,hydrodynamic diameter; DI H_2_O, deionized water; FBS, fetal bovine serum; HS, horse serum; PdI, polydispersity index; ρ_agg_, effective density; RPMI, Roswell Park Memorial Institute medium; SAGM, small airway epithelial cell growth medium; σ, specific conductance; ζ, zeta potential. Values represent the mean (± SD) of a triplicate reading.					

### *In Vitro* and *in Vivo* Dosimetric Considerations

To express *in vivo* and *in vitro* doses on the same scale, we used the dosimetric approach recently developed by the authors (Demokritou et al. 2013). In summary, the multiple-path particle dosimetry model (MPPD2) (Anjilvel and Asgharian 1995) was used to calculate the deposition mass flux in the human lung (micrograms per square meter minute) and the deposited PEP mass per area (micrograms per square meter) following inhalation exposure to PEPs for a given amount of time. Table S1 (see Supplemental Material) summarizes the parameters used for the MPPD2 simulations, including both the airborne nanoparticle size distribution values (count median diameter, geometric standard deviation, particle mass concentration) and the human breathing parameters of a resting individual (tidal volume, breathing frequency, inspiratory fraction, pause fraction, functional residual capacity, head volume, breathing route). The calculated mass per area deposited in the lung obtained from the model is the equivalent mass per area (micrograms per square meter) that must be delivered to cells *in vitro* (mass deposited *in vitro*).

Because of the particokinetics of the PEP-media suspension that define the settling rate, the mass that is delivered to cells *in vitro* is not necessarily equal to the administered mass. Therefore, the fraction of the administered particle mass that is deposited on the cells as a function of *in vitro* exposure time (f_D_) must be calculated in order to match the *in vivo* lung-deposited dose estimated by the MPPD2 model. The f_D_ as a function of *in vitro* exposure time is calculated using the hybrid volumetric centrifugation method–*in vivo* sedimentation, diffusion and dosimetry (VCM–ISDD) method (Cohen et al. 2014b; DeLoid et al. 2014; Pal et al. 2015) that was recently developed by the authors. The mean media-formed agglomerate d_H_ and the VCM-measured effective density of formed agglomerates (DeLoid et al. 2014) were input to the VCM–ISDD fate and transport numerical model in order to estimate the f_D_ as a function of time. For more details, please refer to the Supplemental Material, “Part A: Dosimetric considerations for *in vitro* testing—example of calculations.”

### Source and Characterization of Control Particles

Gas metal arc–mild steel welding fumes (MS-WF) were used as control material in the study and were provided by J. Antonini from the National Institute for Occupational Health (NIOSH). The sample, with a count mean diameter of 1.22 μm, was generated as described in Antonini et al. (1999) and has been shown to induce toxicity in the lungs of rodents (Antonini et al. 2012; Sriram et al. 2012; Zeidler-Erdely et al. 2011). Its Brunauer-Emmett-Teller (BET; BET Surface Area Analyzer, Quantachrome) specific surface area was 48.2 m^2^/g, and its equivalent primary particle diameter was estimated at 23.8 nm. Amorphous silicon dioxide (SiO_2_) was generated in-house using the Harvard versatile engineered nanomaterial generation system (VENGES) as previously described (Demokritou et al. 2010; Sotiriou et al. 2012) and had a BET measured primary particle diameter of 14.7 nm. Both materials were used as controls owing to the extensive toxicological data for these materials that are available in the literature at present.

### Cell Culture

Immortalized human monocytic cells (THP-1, American Type Culture Collection) were cultured in Roswell Park Memorial Institute medium (RPMI) 1640 supplemented with 10% fetal bovine serum (FBS). Small airway epithelial cells (SAECs) were obtained from NIOSH and were cultured in serum-free small airway epithelial cell growth medium (SAGM) with the addition of multiple supplemental growth factors provided by the manufacturer (Lonza Inc.). TK6 human lymphoblastoid cells were maintained in RPMI 1640 with L-glutamine supplemented with 10% horse serum (HS). It should be noted that the TK6 lymphoblast cell line used here may not be directly physiologically relevant to lung toxicology. However, this cell line has been used historically to evaluate genotoxicity owing to its increased sensitivity for DNA damage assessment, in particular when performing the comet assay (Bajpayee et al. 2013; Kimura et al. 2013). Here, TK6 cells were used to rank PEPs in terms of DNA damage potential on the basis of this record of usefulness. All media were supplemented with 1% penicillin-streptomycin. Generic cell culture protocol consisted of growing cells in an incubator (37°C, 5% CO_2_) in 25- or 150-cm^2^ flasks, replacing media every 2–3 days and passaging before confluence. Before exposure to the toxicants, THP-1 cells were differentiated into macrophages (Daigneault et al. 2010).

### Cellular Assays

Various cellular assays were used to assess biological mechanisms. All experiments were performed in triplicate.

*Cellular membrane integrity.* After being exposed to the test particles, cells were evaluated for viability using the CytoTox-One Homogenous Membrane Integrity Assay (Promega). This assay estimates the number of nonviable cells present after exposure by measuring the activity of lactate dehydrogenase (LDH) leaked from the cell.

*ROS production.* After 23.5 hr of particle exposure, dihydroethidium (DHE) was added to each treatment well to prepare a 5-μM suspension of the cells and incubated for 30 min. Fluorescence measurements were taken immediately using a fluorescence microplate reader (Molecular Devices) at an excitation wavelength of 518 nm and an emission detection wavelength of 605 nm. Hydrogen peroxide was used as a positive control in this assay; although these measurements are not shown in the figure, they were used in the calculations to normalize the data.

*Autofluorescence of ENMs pertaining to both cellular membrane integrity and ROS assays.* Autofluorescence of ENMs and media can cause interference with fluoroscopic bioassays (Doak et al. 2009; Holder et al. 2012; Monteiro-Riviere et al. 2009), and control experiments with particles only and with media only must be included in the measurement to consider particle/media interference. We performed such experiments in this study to estimate potential nanoparticle interference/absorption in the LDH and ROS assays, and we measured the fluorescence intensity of the particles suspended in media. The intensity was minimal and was similar to that of the media-only control for both bioassays; therefore, this value was included in the calculations (results not shown).

*DNA damage.* To assess the potentially genotoxic properties of PEPs, the high throughput Nano-CometChip assay (recently developed by our group) was used to measure DNA double-stranded breaks on TK6 cells following a 4-hr exposure to particles, as described in Watson et al. (2014).

*Epigenetic analysis.* Assays were performed to evaluate DNA methylation patterns on SAECs exposed to PEPs (administered doses of 0.5 and 30 μg/mL) for 24 hr. In more detail:

Methylation of transposable elements. RNA and DNA were extracted simultaneously from SAECs using an AllPrep Mini Kit (Qiagen) according to the manufacturer’s protocol. Analyses of methylation and of expression of transposable elements open reading frame 1 (ORF1), ORF2, and Alu were performed as reported previously (Lu et al. 2015). Briefly, 500 ng of gDNA was treated with 0.5 U of SmaI, HpaII, HhaI, AciI, and BstUI enzymes in 1X CutSmart buffer. The resulting digested DNA was analyzed by quantitative real-time PCR (qRT-PCR) using 2 ng DNA per reaction and SYBR Select Master Mix (Life Technologies). Primers are listed in Supplemental Material, Table S2.

Expression of transposable elements. cDNA was synthesized from 1 μg RNA using a High-Capacity Reverse Transcription Kit (Life Technologies). qRT-PCR was performed using 10 ng cDNA per reaction and SYBR Select Master Mix on a ViiA 7 instrument (Life Technologies). Primers are listed in Supplemental Material, Table S2. Expression was calculated using the ΔΔCt method and normalized to the internal control GAPDH.

LINE-1 copy number analysis. LINE-1 copy number was assessed as previously described (Miousse et al. 2014b). Briefly, LINE-1 ORF1 was amplified from 10 ng of gDNA by qRT PCR. The FAM/ZEN-conjugated primers containing the probe sequence (Integrated DNA Technologies) are shown in Supplemental Material, Table S3. The relative abundance of the target in gDNA was normalized to 5S ribosomal DNA using the ΔΔCt method.

### Cytokine and Chemokine Analysis

Supernatants from treated SAECs were assayed by Eve Technologies Corporation, which used a Human Primary Cytokine Array/Chemokine Array 41-Plex Panel (Millipore) according to the manufacturer’s protocol.

### Statistical Analysis

Statistical analyses were performed using GraphPad Prism 6.0 (GraphPad Software, Inc.). Comparisons among all cellular parameters after exposure were evaluated for statistical significance using one-way analysis of variance and Tukey’s multiple comparison test. A *p-*value < 0.05 was considered significant. Experiments were performed in triplicate.

## Results

### PEP Dispersion and Characterization

Supplemental Material, Figure S1, shows the hydrodynamic diameter of both PEPs and MS-WF plotted as a function of delivered sonication energy (DSE). As the DSE increases, the dynamic light scattering (DLS)-measured d_H_ decreases toward a marginal state of minimal agglomeration. The DSE_cr_ for PEPs (PM_0.1_) was 514.29 J/mL. Similarly, the DSE_cr_ for MS-WF was 400 J/mL. The DSE_cr_ for SiO_2_ was 242 J/mL and was obtained from a previous publication (Cohen et al. 2013).

Table 1 summarizes the particle colloidal properties in DI H_2_O and in different types of biological media; these properties include the DLS-measured hydrodynamic diameter (d_H_), the zeta potential (ζ), the polydispersity index (PdI), the specific conductance (σ), and the pH. The d_H_ of PEPs (PM_0.1_) suspended in DI H_2_O was lower than that of PEPs suspended in cellular media. PEPs (PM_0.1_) had a d_H_ of 178.3 nm in DI H_2_O, which increased to > 200 nm when they were dispersed in media. This finding is in accord with other results in the literature (Cohen et al. 2013) because it is expected that the presence of proteins in media induces the formation of a thicker protein corona on particle agglomerates. MS-WF suspended in DI H_2_O had a d_H_ of 2,197 nm, which decreased in media to values ranging from 1,502 to 1,878 nm. Lastly, the d_H_ of silica was 142.5 nm in DI H_2_O and 114.6–207.7 nm in media. The observed zeta potential values were strongly negative for PEPs in DI H_2_O (–20.6 mV) and became less negative in media. MS-WF and SiO_2_ had positive zeta potentials in both DI H_2_O and media. In addition to obtaining d_H_ measurements, we evaluated the colloidal size stability of particle suspensions for 24 hr. The d_H_ of PEPs, SiO_2_, and MS-WF suspended in SAGM remained fairly stable for up to 24 hr.

Additionally, the VCM-measured effective density of PEPs ranged from 1.19 to 2.39 g/cm^3^ in different cellular media, whereas the effective densities of the other materials were approximately 1.2 g/cm^3^ (SiO_2_) and 1.37 to 1.56 g/cm^3^ (MS-WF) (Table 1). It should be noted that the effective density and size of formed agglomerates are important determinants of their fate and transport in *in vitro* systems, and these properties define settling rates and dosimetry *in vitro* (DeLoid et al. 2014; Cohen et al. 2013; Pal et al. 2015).

### **Dosimetric Considerations for** *in Vitro* **Testing**

The delivered-to-cell dose at a given exposure time point may not always be the same as the administered dose (Cohen et al. 2013). We used the recently developed Harvard *in vitro* dosimetry methodology (Cohen et al. 2014b) to calculate the fraction of administered particles that deposited on the cells located at the bottom of the treatment well as a function of time (see Supplemental Material, Figure S2). As expected, some materials settled faster than others. For instance, all of the administered MS-WF mass, suspended in either RPMI/10% FBS or SAGM, was deposited on the cells in ≤ 2 hr. In contrast, only approximately 35% and 100% of the administered dose of silica suspended in RPMI/10% FBS and SAGM, respectively, actually reached the bottom of the well in 24 hr. Interestingly, with the same exposure duration, 100% and 51.8% of the administered dose of PEPs suspended in SAGM and RPMI/10% FBS, respectively, were deposited on the cells, which translated to f_D_ values of 1.00 and 0.518, respectively. The estimated deposited mass of administered particles for all PEP doses and exposure times is summarized in Table 2 (see Supplemental Material, Table S4, for estimated deposited masses for SiO_2_ and MS-WF).

**Table 2:** *In vitro* doses of PEPs and the corresponding consumer inhalation exposure duration.

Administered dose (cells)^*a*^ (μg/mL)	SAEC		THP-1	
	Delivered dose (cells)^*a*^ (μg/mL)	Corresponding consumer inhalation exposure duration to PEPs (hr)^*b*^	Delivered dose (cells)^*a*^ (μg/mL)	Corresponding consumer inhalation exposure duration to PEPs (hr)^*b*^
0.5	0.5	15.0	0.26	7.8
5	5	75.2	2.6	39.0
10	10	150.4	5.2	77.9
20	20	300.7	10.4	155.8
30	30	451.1	15.6	233.7
40	40	601.4	20.8	311.5
100	100	1503.6	52.0	778.9
^***a***^*In vitro*–administered and delivered doses were based on a 24-hr *in vitro* exposure. ^***b***^Calculations of the corresponding consumer inhalation exposure duration (hours) were based on the added values of deposition mass flux (μg/m^2^ • min) in the various human airways, excluding head airways: the conducting zone (generations 0 to 16) and the transitional and respiratory zones (generations 17 through 23).				

Additionally, to bring *in vitro* and *in vivo* doses to the same scale, the deposition mass flux of PEPs in a human lung was determined to be 1.732 μg/m^2^ • min using the MPPD2 model. This calculated mass flux was then used to back-calculate the duration of inhalation exposure to PEPs corresponding to the range of administered doses used in this study (summarized in Table 2). Based on dosimetric calculations for THP-1 monocytes, the lowest *in vitro*–administered dose of PEPs was consistent with an inhalation exposure lasting for 7.8 hr of printing, whereas the highest administered dose (100 μg/mL) corresponded to hundreds of hours of exposure. The wide range of human exposures corresponding to laser printer emissions evaluated here makes the doses relevant for individuals in both occupational and consumer settings. The majority of the inhaled PEPs would deposit in the respiratory bronchioles and distal alveoli (see Supplemental Material, Figure S3). Approximately 31% of inhaled PEPs would deposit in the tracheobronchial region, and 18.4% would deposit in the head region. Although the cell lines used in this study represent the types of cells that are located in the lower respiratory area, it should be noted that the upper airways are an equally interesting target.

### Effects of PEPs on Cell Viability

**Figure 1:**
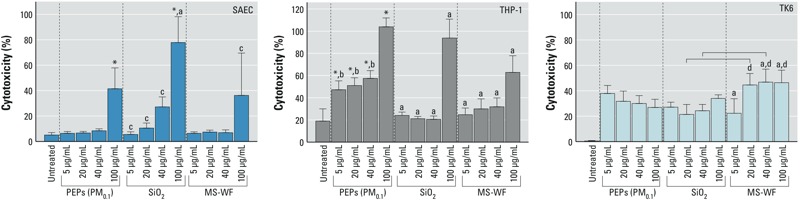
Percent cytotoxicity of cells determined using the LDH assay following exposure to PEPs (PM_0.1_), SiO_2_, and MS-WF on three human cell lines (SAEC, small airway epithelial cell; THP-1, monocytic cell line; TK6, lymphoblast cell line). All values are represented as the mean ± SE. *p < 0.05, values significantly different from those for untreated cells: a,****PEPs (PM_0.1_) dose-matched; b, PEPs (PM_0.1_) 100 μg/mL; c, SiO_2_ 100 μg/mL; d, MS-WF 5 μg/mL treatment groups. Bar represents a significant difference in measurements across the treatment groups with *p* < 0.05.

The cellular membrane integrity of all three human cell lines decreased following exposure to PEPs. Figure 1 illustrates results from the lactate dehydrogenase assay, showing the percent cytotoxicity of each treatment at various administered doses. In particular, SAECs experienced > 40% cell death after exposure to PEPs (PM_0.1_, 100 μg/mL administered dose) when compared with untreated cells. Macrophages (THP-1 cells) exposed to PEPs (PM_0.1_) exhibited a significant increase in cell death in a dose–response manner. This response was greater than that shown with MS-WF or SiO_2_ treatment; MS-WF is known to be cytotoxic (Antonini et al. 1999, 2012; Zeidler-Erdely et al. 2011). Last, cytoxicity to human lymphoblasts (TK6 cells) decreased with increasing exposure to PEPs (PM_0.1_), although differences among dose groups were not significant.

### Effects of PEPs on ROS Production

**Figure 2:**
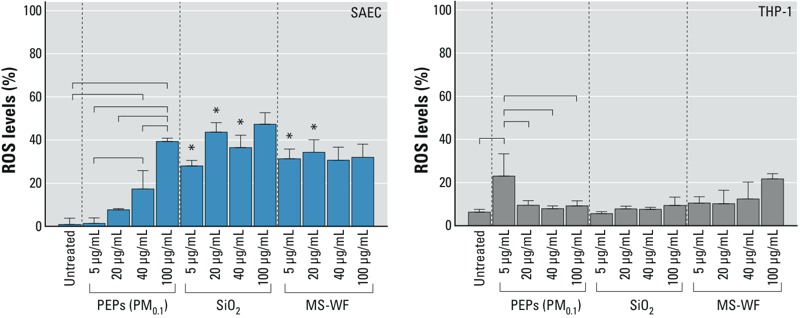
Percent increase of reactive oxygen species compared with that in untreated control cells; measured in supernatant from SAECs and THP-1 cells following a 24-hr exposure to PEPs (PM_0.1_), SiO_2_, and MS-WF. All values are represented as the mean ± SE. *Significantly different (*p* < 0.05) from PEPs (PM_0.1_), dose-matched treatment group. Bar represents a significant difference in measurements across the treatment groups with *p* < 0.05.

To evaluate the potential of PEPs to induce ROS production in epithelial cells (SAECs) and macrophages (THP-1 cells), two types of cells that are in direct contact with inhaled foreign material, the levels of superoxide ions were measured. Figure 2 presents the results from the DHE fluorescence assay for each treatment at various doses and shows the contrasting responses in both cell lines. A clear dose–response relationship was observed in SAECs treated with PEPs. Although MS-WF and SiO_2_ also enhanced ROS production in SAECs, dose dependence was not observed. The level of ROS production in SAECs exposed to PEPs (100 μg/mL administered dose) was similar to that in SAECs exposed to an administered dose of 100 μg/mL MS-WF or SiO_2_. Macrophages (THP-1 cells) displayed elevated superoxide levels following exposure to PEPs (5 μg/mL administered dose), but higher doses did not induce ROS production. Treatment with PEPs (5 μg/mL) was more potent in stimulating ROS release than SiO_2_ or MS-WF at the same administered dose.

### Effects of PEPs on Inflammatory Mediator Secretion

**Figure 3:**
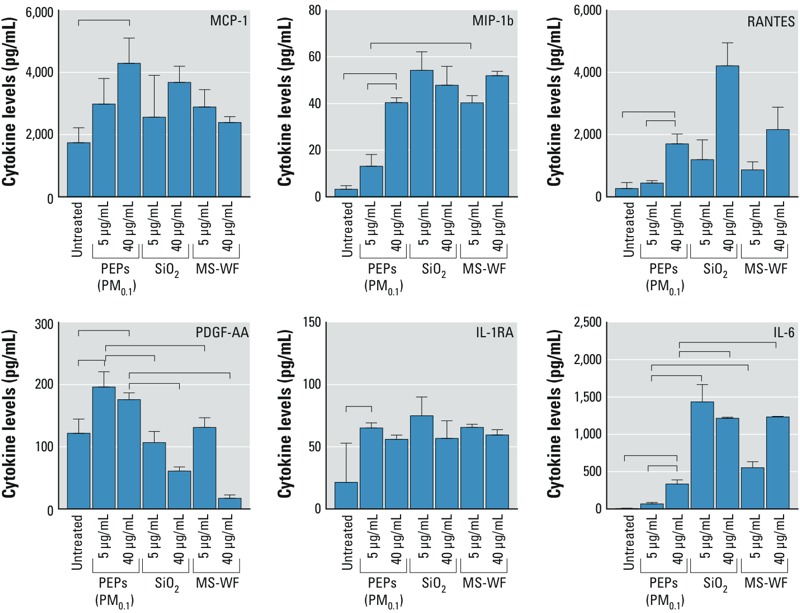
Measured levels of cytokines and chemokines in supernatant of SAECs exposed to PEPs, SiO_2_, and MS-WF for 24 hr. All values are represented as the mean ± SE. Bar represents a significant difference in measurements across the treatment groups with *p *< 0.05.

Cytokine/chemokine release plays an important role in the regulation of an immune response toward pathogens or injury (Lacy and Stow 2011). In order to evaluate the effects of PEPs on such biological reactions, levels of a wide variety of these mediators were measured in SAECs following a 24-hr exposure to PEPs (5 and 40 μg/mL administered doses). Of the 41 measured cytokines/chemokines, 6 of them, namely monocyte chemotactic protein (MCP)-1, macrophage inflammatory protein (MIP)-1b, platelet-derived growth factor (PDGF)-AA, interleukin (IL)-1RA, IL-6, and RANTES, were significantly increased in SAECs exposed to PEPs (PM_0.1_) (Figure 3). After exposure to PEPs (40 μg/mL administered dose), the levels of MCP-1, MIP-1b, RANTES, PDGF-AA, and IL-6 were significantly higher in treated cells than in the controls. In addition, there was a significant difference in the levels of MIP-1b and IL-6 in SAECs exposed to both doses of PEPs (5 and 40 μg/mL). Exposure to PEPs (5 μg/mL administered dose) led to a significant rise in IL-1RA and PDGF-AA secretion in treated versus untreated cells.

### Effects of PEPs on Genotoxicity in TK6 Lymphoblasts

To evaluate the genotoxic potential of PEPs, a DNA damage assessment was performed on human lymphoblasts (TK6 cells), which are genetically sensitive to chemical exposure (Ayres et al. 2006; Kimura et al. 2013). The results from the Nano-CometChip assay indicate that PEPs did not inflict significant DNA damage on the lymphoblasts (see Supplemental Material, Figure S4). Similarly, neither of the other types of particles (SiO_2_, MS-WF) induced single-stranded DNA damage in the treated cells.

### Effects of PEPs on Global and TE-Associated DNA Methylation

**Figure 4:**
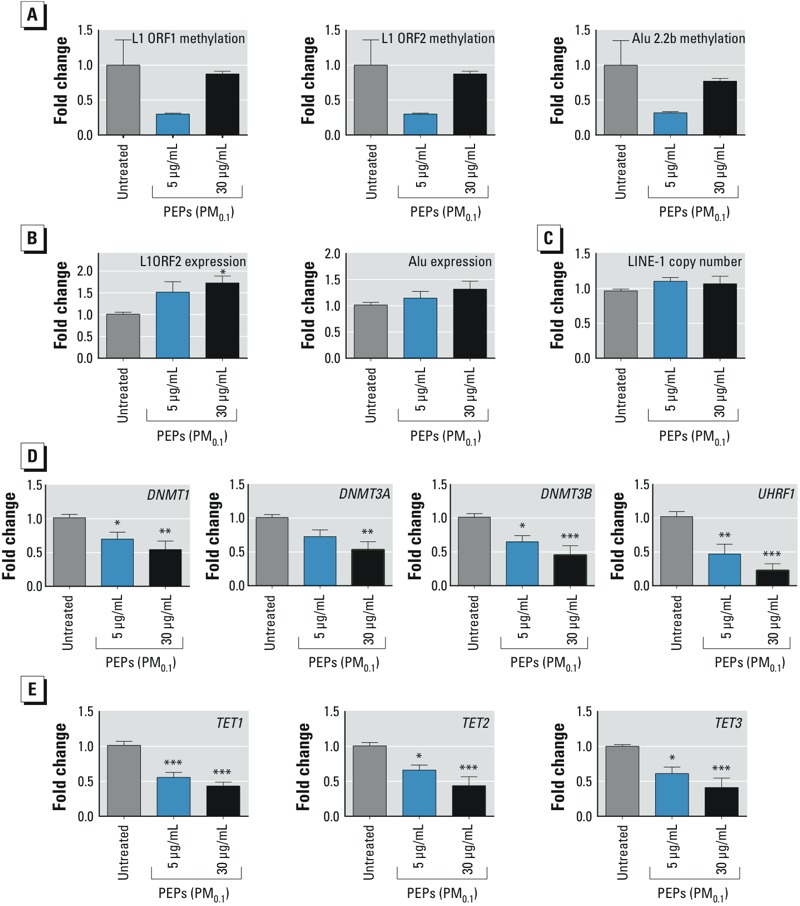
DNA methylation in SAECs exposed to PEPs for 24 hr compared with that in the untreated control. (*A*) Fold change in 5-meC in TEs; (*B*) mRNA expression of TEs; (*C*) LINE-1 copy number; (*D*) expression of DNMTs and accessory protein *UHRF1*. (*E*) Expression of methylcytosine deoxygenases (*TET1-TET3*) in SAECs exposed to PEPs for 24 hr. All values are represented as the mean ± SE. *p< 0.05. **p< 0.01. ***p< 0.001.

L1 repetitive elements comprise approximately 17% of the human genome and are heavily methylated; therefore, the methylation status of L1 elements is generally accepted as a surrogate biomarker for global DNA methylation (Miousse et al. 2015). Therefore, to investigate whether short-term exposure to PEPs can affect global DNA methylation, the methylation patterns of both L1 open reading frames (ORF1, ORF2) were evaluated. A loss of DNA methylation after exposure to PEPs (0.5 μg/mL administered dose) was observed in ORF1 and ORF2, although it was not statistically significant (*p-*value 0.09 for both cases) in treated versus untreated cells. No significant changes in DNA methylation were detected after exposure to an administered dose of 30 μg/mL PEPs (Figure 4A).

*Alu* elements are another group of TEs that are highly abundant in the human genome (comprising ~ 10%); these correspond to SINE elements in mice and can be affected by exogenous stressors (Rudin and Thompson 2001). Thus, we addressed whether the methylation of *Alu* elements was also affected by PEPs by examining the AluYb11 subfamily belonging to the SINE1/7SL family of evolutionary-recent *Alu* elements. Based on comparisons with untreated cells, treatment with 0.5 μg/mL (administered dose) PEPs led to an approximately 70% decrease in *Alu* methylation, although not statistically significant, whereas exposure to 30 μg/mL (administered dose) PEPs did not affect methylation of *Alu* (Figure 4A).

### Effects of PEPs on TE Expression

Methylation of TEs is a key mechanism in preventing their aberrant expression, and hypomethylation of TEs is often associated with their reactivation due to various environmental stressors (Koturbash et al. 2011; Rudin and Thompson 2001). Therefore, the expression of L1 ORF2 was measured because this region is critical for the activation and retrotransposition of L1.

After treatment with 0.5 and 30 μg/mL PEPs (administered doses), expression of L1 ORF2 was 1.5 and 1.7 times higher, respectively, than in untreated controls, and a significant increase in expression occurred at the higher dose (Figure 4B). Transcriptional activation of L1 may result in retrotransposition on the “copy-paste”–based mechanism, thus increasing the L1 copy number in the genome. Therefore, the L1 ORF1 copy number was analyzed; however, no significant differences were identified (Figure 4C). Although not statistically significant, the expression of *Alu* increased by 15% and 32% after exposure to 0.5 and 30 μg/mL of PEPs, respectively (Figure 4B).

### Effects of PEPs on DNA Methyltransferase and Methylcytosine Dioxygenase Expression

To further investigate the mechanisms of observed global and TE-associated DNA hypomethylation, we investigated the expression of DNA methyltransferases, key enzymes needed for the establishment and maintenance of normal methylation patterns. Compared with untreated cells, a significant and dose-dependent reduction in the expression of all three DNA methyltransferases (DNMT1, DNMT3A, DNMT3B) was detected after PEP exposure (Figure 4D). Additionally, the expression of UHRF1, the protein that recruits DNMT1 to hemimethylated DNA sites, was significantly reduced in a dose-dependent manner after PEP exposure. A significant and dose-dependent reduction in the expression of all three methylcytosine dioxygenases (TET1–TET3) was observed (Figure 4E).

## Discussion

The objective of this study was to evaluate the potential toxicity of various doses of PEPs in human small airway epithelial cells (SAECs), macrophages (THP-1 cells) and lymphoblasts (TK6 cells). Using doses that approximate those associated with inhalation exposures, we measured cell membrane integrity, ROS production, inflammatory responses, DNA integrity, and epigenetic changes. Because the aim of the study was to understand the biological response of cells following exposure to PEPs, we administered doses at both the low (0.5 μg/mL) and high (100 μg/mL) ends of the spectrum. Low-end doses correspond to exposure durations at levels experienced by consumers (e.g., 8 hr of exposure to PEPs), whereas high-end doses correspond to the accumulation of hundreds of hours of exposure. It must be noted that the dosimetric approach presented herein may only be appropriate for short-term human exposures on the order of a few days. Equating lifetime or multiyear accumulations of PEP mass in alveolar regions with *in vitro* bolus delivery ignores differences in exposure dose and rate. These differences may span orders of magnitude and affect clearance mechanisms, thereby producing misleading results. Doses on the high end of the spectrum should only be considered as the limit of an *in vitro* investigation and only when a wide range of doses, including low-end doses, is used. Therefore, the high administered dose of 100 μg/mL was included to obtain the full spectrum of dose–response relationships.

This publication is part of a series of companion papers evaluating the toxicological profile of PEPs. First, the PEGS exposure platform developed by our group (Pirela et al. 2014) was used to rank and evaluate eleven commonly used printers on the basis of their PM emission profiles. Second, the complete physicochemical and morphological properties of several toner powders and PEPs were thoroughly assessed (Pirela et al. 2015), thereby establishing that toner powders contain ENMs that become airborne during printing (consumer use). Third, it was shown that low-level exposure to PEPs (PM_0.1_, PM_2.5_) led to significant biological outcomes in an *in vitro* alveolar–capillary coculture model (Sisler et al. 2015). Further investigation of paracrine signaling by epithelial and endothelial cells is of utmost significance because cellular communication between these critical cell lines may play a major role in the pathogenesis of various pulmonary disorders.

Here, we investigated the toxicological potential of the smallest-size fraction (PM_0.1_) of PEPs from a laser printer emitting 1.26 particles/cm^3^ [printer B1 in previous publications (Pirela et al. 2014, 2015)] using a monocell culture experimental design. Because the alveolar epithelium has direct contact with inhaled nanoparticles (Don Porto Carero et al. 2001), and because the alveolar macrophages are the first responders to foreign particles in the lung, we exposed these cells to various concentrations of PEPs and observed the responses to these particles. The results showed that both the epithelial cells (SAECs, at a 100-μg/mL delivered dose) and the macrophages (THP-1 cells, at a 2.59-μg/mL delivered dose) were negatively affected by treatment with PEPs and experienced > 40% cell death. Of note, macrophages (THP-1 cells) seem to be particularly sensitive to exposure to PEPs, which proved to be more toxic than a known pulmonary irritant (MS-WF). This finding is in accord with a study by Khatri et al. (2013b), which showed subtle dose–response changes in the viability of THP-1 cells and SAECs following a 24-hr exposure to particles sampled from a photocopier center, which had a similar chemical composition to that of PEPs. As previously shown in a companion study, SAEC viability following exposure to PEPs (PM_0.1_) was lower than that after exposure to PEPs (PM_2.5_) at a delivered dose of 2.5 μg/mL, indicative of the greater potency of PEPs (PM_0.1_) (Sisler et al. 2015).

In summary, these results indicate significant cytotoxicity of PEPs, which could lead to defects in the normal function of these cells; macrophages could be particularly affected because they primarily engulf foreign materials. Cytotoxicity of PEPs to macrophages could impair their clearance mechanism, affect cellular crosstalk, and influence the innate immune response. The amount of cytotoxicity observed in the tested cell lines at doses corresponding to inhalation exposures ranging from 7.8 to 1,500 hr further intensifies recent concerns that PM emitted from laser printers can trigger a response in the distal alveolar region, where the majority of the inhaled particles deposit. The toxicity of PEPs might be attributable to their complex chemical composition, which includes various nanosized metals/metal oxides that have already been shown to produce detrimental effects in various *in vitro* and *in vivo* studies. The toxicological outcomes of these studies include decreased cell viability, increased production of ROS, and agglomeration of internalized particles due to exposure to various ENMs (e.g., titania, silica, ceria, iron oxide, silver) (Cohen et al. 2014a; Demokritou et al. 2013; L’Azou et al. 2008; Watson et al. 2014). In summary, the vulnerability of respiratory bronchioles and alveoli to exogenous materials highlights the necessity of understanding the amount of damage PEPs can cause to consumers’ respiratory systems and to other organ systems (i.e., cardiovascular, immunological) without disregarding susceptible individuals. It should also be noted that our recent studies using photocopy center–sampled particles indicated that those particles may produce adverse responses in the lung physiology of individuals who are exposed even at relatively low doses (Khatri et al. 2013a, 2013b; Pirela et al. 2013, 2015).

Another relevant parameter used to evaluate the adverse effects of exposure to airborne PM in general is cytokine secretion. The expression of these chemical messengers was evaluated in SAECs to quantify the inflammatory response to PEPs. The results showed that exposure to PEPs (PM_0.1_) significantly upregulated the expression of MCP-1, MIP-1b, PDGF-AA, IL-1RA, IL-6, and RANTES. These mediators are critical to the innate immune process, which recruits leukocytes to sites of injury/inflammation (Hayden et al. 2009; Ritter et al. 2005). In a companion study that used an epithelial–endothelial cell coculture system (Sisler et al. 2015), increases in IL-6 and MCP-1 were observed following low-level exposure to PEPs (PM_0.1_, PM_2.5_). These results are in accord with those of a study by Setyawati et al. (2013), in which endothelial cells treated with nanotitania reacted in a non–receptor-mediated mechanism and triggered endothelial cell leakiness. Similarly, macrophages, primary nasal epithelial cells, and SAECs exposed to various doses of photocopy center–sampled particles exhibited elevated secretion of various cytokines, namely GM-CSF, IL-1b, IL-6, IL-8, TNFα, and VEGF (Khatri et al. 2013b). Furthermore, these cytokines were also overexpressed in nasal lavage from human volunteers exposed to copy-center particles for 6 hr (Khatri et al. 2013a). In particular, MCP-1 is a known monocyte chemoattractant that is produced by monocytes and macrophages due to stressors (e.g., oxidative damage, cytokines, growth factors). This chemokine regulates the migration and infiltration of monocytes, memory T cells, and natural killer cells to injury sites, which mainly leads to differentiation of precursor cells into Th2 cells. Therefore, modifications in MCP-1 levels may indicate that exposure to PEPs can affect monocyte/macrophage recruitment in the lung for phagocytosis of invading pathogens (Deshmane et al. 2009). Moreover, expression of MCP-1 can in turn contribute to an increase in the levels of IL-6, which blocks apoptosis. A study by Liu et al. (2007) found that MCP-1 mediated fibroblast survival by elevating IL-6 levels via the IL-6/STAT3 signaling pathway. Consequently, apoptosis of fibroblasts was inhibited, which resulted in continued lung fibrosis. Additionally, RANTES has been found to be strongly upregulated in response to asbestos exposure, a cause of malignant mesothelioma (Comar et al. 2014). Other cytokines that were shown to be significantly affected in both pleural fibrosis and malignant mesotheliomas include IL-6, IL-1b, and IL-8, possibly through inflammasome activation (Hillegass et al. 2013). These same cytokines were observed to be affected after exposure to PEPs (Sisler et al. 2015). Comparable changes in the expression of TNFα, IL-1a and IL-1b, IL-6, MCP-1, and PDGF-AA were observed in mice exposed to multiwalled carbon nanotubes (Dong et al. 2015). Thus, Dong et al. (2015) concluded that such exposure was associated to an inflammatory and fibrotic response in the lung. However, more mechanistic studies investigating upstream effectors of the common process underlying these changes in cytokine expression, such as activation of NF-κB, are needed to enhance our understanding of inflammatory responses due to PEP exposure. We plan to perform in-depth toxicological assessments to better understand the observed inflammatory responses and to report our findings in a future companion paper.

In addition to the inflammatory responses, an increase in superoxide levels was evident in epithelial cells after treatment with PEPs. Similar to our results, Sisler et al. (2015) observed an increment of ROS in endothelial cells after epithelial cells were exposed to low doses of PEPs in a coculture platform. This result was not observed for macrophages (THP-1 cells) treated with PEPs, whose cytotoxicity is almost 100% at the high dose of 100 μg/mL. However, at the same dose, the macrophages produced small amounts of ROS, which suggested that the observed cytotoxicity might be mediated independently of ROS. Potential mechanisms include direct activation of caspase-mediated apoptosis, as observed in macrophages treated with zinc oxide nanoparticles (Wilhelmi et al. 2013); surface reactivity effects (Fröhlich et al. 2009); or the HIF pathway (Nyga et al. 2015). More detailed mechanistic studies are needed to better understand the observed cytotoxicity. Overall, our findings are consistent with those of studies showing an increase in extracellular levels of ROS and the concomitant downregulation of antioxidant levels after treatment with various doses of currently available ENMs such as ceria, titania, and cobalt (Mittal and Pandey 2014; Wan et al. 2012; Zarogiannis et al. 2013).

Furthermore, the observed elevated levels of oxidation and inflammation prompted us to use the newly developed high-throughput Nano-CometChip assay (Watson et al. 2014) to assess DNA damage following exposure to PEPs. Human lymphoblasts (TK6 cells) exposed to various doses of PEPs did not exhibit DNA damage, unlike previous *in vitro* studies of genotoxicity in human epithelial lung cells, which revealed formation of micronuclei and other characteristic injuries pertaining to DNA damage in cells exposed to printer-emitted PM and toner powder (Gminski et al. 2011; Tang et al. 2012). Similarly to our findings, the results of a study by Khatri et al. (2013b), which used the comet assay, revealed that treatment of macrophages with copy center–sampled particles did not cause significant DNA damage. The lack of single-stranded DNA damage observed after exposure to PEPs may indicate the possibility of double-stranded DNA damage or another mechanism responsible for the observed increase in cell death. It is important to note that heterogeneity in the chemical composition of PEPs, which was well-documented in our earlier study (Pirela et al. 2015), may explain differences in PEP genotoxicity. The relationship of variability in the chemical makeup of PEPs to their genotoxicity deserves further research.

In the present study, the ability of PEPs to affect the cellular epigenome was demonstrated. Specifically, we found preliminary evidence that short-term exposure to PEPs may result in altered DNA methylation in SAECs, thus affecting the methylation status of two of the most abundant TEs in the human genome—L1 and *Alu*—that together comprise almost 30% of the genome. Future studies are needed to confirm these assumptions.

DNA methylation is the key mechanism that prevents aberrant transcriptional activity of TEs (Smith et al. 2012). Loss of DNA methylation within TEs often results in their transcriptional activation (Koturbash et al. 2011; Rudin and Thompson 2001). Reactivation of TEs can, in turn, result in retrotransposition and lead to genomic instability and development of diseases, including cancer. In the present study, the expression of L1 ORF2 was elevated in a dose-dependent manner following exposure to both concentrations of PEPs tested. Similar trends were observed for *Alu* elements, although the results were not statistically significant. This transcriptional activation, however, did not result in potential retrotransposition events because no significant increase in L1 copy number was identified after exposure to PEPs. It is possible that the time of exposure was not sufficient for detectable L1 retrotransposition to occur. Indeed, a recent study on chemical exposure and L1 retrotransposition reported L1 mobilization in cell culture after 120 hr of exposure (Terasaki et al. 2013). Further studies using longer exposure times are clearly needed to determine the L1 retrotransposition abilities of PEPs.

In the present study, we detected a dose-dependent decrease in the expression of DNA methyltransferases caused by exposure to PEPs. These enzymes are essential for proper maintenance of DNA methylation. A loss of DNA methyltransferases *in vitro* was previously reported after short-term exposure to PM (Miousse et al. 2014a) and nano-SiO_2_ particles (Gong et al. 2010); this loss was also associated with alterations in global and TE DNA methylation. The observed downregulation of DNA methyltransferases after exposure to PEPs may have detrimental effects on the levels of DNA methylation beyond the 24-hr time point used in the present study. Importantly, we have provided evidence that hypomethylation of TEs and loss of expression of DNA methyltransferases may occur after exposure to low, environmentally relevant doses (0.5 μg/mL) of PEPs. The mechanisms of these alterations may be associated with metals present in PEPs. In their vast majority, metals are weak mutagens, but they can negatively affect the enzymatic activity of DNA methyltransferases (Fragou et al. 2011). Furthermore, the generation of ROS, associated with metals present in PEPs, may compromise the normal redox status, alter glutathione content, and affect one-carbon metabolism pathways (Koturbash et al. 2012). Hypomethylation may also be mediated by decreased levels of UHRF1, which specifically interacts with DNA methyltransferases and hemimethylated sites on DNA (Ehrlich and Lacey 2013). The exact mechanisms of PEP-associated epigenotoxicity, however, still need to be determined. The loss of TE methylation was not associated with increased function of the methylcytosine deoxygenases that regulate hydroxymethylation, the pathway involved in DNA demethylation (He et al. 2011; Ito et al. 2011). Further studies will be needed to delineate the exact effects of exposure to PEPs on the expression of 5-hmC and TET, especially with regard to studies indicating a loss of 5-hmC TET in numerous diseases, including cancer (Jin et al. 2011; Li et al. 2011).

In summary, exposure to PEPs appears to trigger an unfavorable biological response in several physiologically relevant cell lines. Increased cell death, oxidative stress, inflammation, and altered methylation are some of the negative effects PEPs may have on the lung, and inhalation of these particles may lead to an increased risk of respiratory disorders in individuals who are exposed to emissions from laser printers.

## Conclusion

The results of the present study indicate that PEPs emitted by laser printers can elicit unfavorable biological responses *in vitro.* Exposure to PEPs at doses corresponding to real-world levels led to significant changes in cell viability, hereditary genetic material changes, generation of ROS, and increases in inflammatory mediators, among other effects. Moreover, the observed dysfunction of the DNA methylation and demethylation machinery associated with the loss of DNA methylation and the reactivation of TEs suggests that exposure to PEPs may have significant effects on the cellular epigenome. The results from this comprehensive battery of toxicological assessments of PEPs are indicative of the cyto- and genotoxic potential of laser printer emissions at doses comparable to those received in current consumer and occupational settings. To investigate the mechanism of toxicity in greater detail, a study on murine responses to PEP exposure via intratracheal instillation and whole-body inhalation is in progress. Taken together, our mechanistically oriented toxicological studies could reveal the biological interactions that occur after exposure to PEPs at doses comparable to those experienced by consumers when they use laser printers.

## Supplemental Material

(826 KB) PDF

## References

[r1] Anjilvel S, Asgharian B (1995). A multiple-path model of particle deposition in the rat lung.. Fundam Appl Toxicol.

[r2] Antonini J, Lawryk N, Murthy G, Brain J (1999). Effect of welding fume solubility on lung macrophage viability and function in vitro.. J Toxicol Environ Health A.

[r3] Antonini JM, Zeidler-Erdely PC, Young SH, Roberts JR, Erdely A (2012). Systemic immune cell response in rats after pulmonary exposure to manganese-containing particles collected from welding aerosols.. J Immunotoxicol.

[r4] Ayres FM, da Cruz AD, Steele P, Glickman BW (2006). Low doses of gamma ionizing radiation increase *hprt* mutant frequencies of TK6 cells without triggering the mutator phenotype pathway.. Genet Mol Biol.

[r5] Baccarelli A, Wright RO, Bollati V, Tarantini L, Litonjua AA, Suh HH (2009). Rapid DNA methylation changes after exposure to traffic particles.. Am J Respir Crit Care Med.

[r6] Bai R, Zhang L, Liu Y, Meng L, Wang L, Wu Y (2010). Pulmonary responses to printer toner particles in mice after intratracheal instillation.. Toxicol Lett.

[r7] Bajpayee M, Kumar A, Dhawan A (2013). The comet assay: assessment of in vitro and in vivo DNA damage.. Methods Mol Biol.

[r8] Cohen J, Deloid G, Pyrgiotakis G, Demokritou P (2013). Interactions of engineered nanomaterials in physiological media and implications for *in vitro* dosimetry.. Nanotoxicology.

[r9] Cohen JM, Derk R, Wang L, Godleski J, Kobzik L, Brain J (2014a). Tracking translocation of industrially relevant engineered nanomaterials (ENMs) across alveolar epithelial monolayers *in vitro*.. Nanotoxicology.

[r10] Cohen JM Teeguarden JG Demokritou P 2014b An integrated approach for the in vitro dosimetry of engineered nanomaterials. Part Fibre Toxicol 11 20; doi:10.1186/1743-8977-11-20 24885440 PMC4024018

[r11] Comar M Zanotta N Bonotti A Tognon M Negro C Cristaudo A 2014 Increased levels of C-C chemokine RANTES in asbestos exposed workers and in malignant mesothelioma patients from an hyperendemic area. PLoS One 9 e104848; doi:10.1371/journal.pone.0104848 25162674 PMC4146505

[r12] Daigneault M Preston JA Marriott HM Whyte MK Dockrell DH 2010 The identification of markers of macrophage differentiation in PMA-stimulated THP-1 cells and monocyte-derived macrophages. PloS One 5 e8668; doi:10.1371/journal.pone.0008668 20084270 PMC2800192

[r13] DeLoid G Cohen JM Darrah T Derk R Rojanasakul L Pyrgiotakis G 2014 Estimating the effective density of engineered nanomaterials for *in vitro* dosimetry. Nat Commun 5 3514; doi:10.1038/ncomms4514 24675174 PMC4038248

[r14] Demokritou P, Büchel R, Molina RM, Deloid GM, Brain JD, Pratsinis SE (2010). Development and characterization of a versatile engineered nanomaterial generation system (VENGES) suitable for toxicological studies.. Inhal Toxicol.

[r15] Demokritou P, Gass S, Pyrgiotakis G, Cohen JM, Goldsmith W, McKinney W (2013). An *in vivo* and *in vitro* toxicological characterisation of realistic nanoscale CeO_2_ inhalation exposures.. Nanotoxicology.

[r16] Demokritou P, Kavouras IG, Ferguson ST, Koutrakis P (2002). Development of a high volume cascade impactor for toxicological and chemical characterization studies.. Aerosol Sci Technol.

[r17] Deshmane SL, Kremlev S, Amini S, Sawaya BE (2009). Monocyte chemoattractant protein-1 (MCP-1): an overview.. J Interferon Cytokine Res.

[r18] Doak SH, Griffiths SM, Manshian B, Singh N, Williams PM, Brown AP (2009). Confounding experimental considerations in nanogenotoxicology.. Mutagenesis.

[r19] Don Porto Carero A, Hoet PH, Verschaeve L, Schoeters G, Nemery B (2001). Genotoxic effects of carbon black particles, diesel exhaust particles, and urban air particulates and their extracts on a human alveolar epithelial cell line (A549) and a human monocytic cell line (THP-1).. Environ Mol Mutagen.

[r20] Dong J, Porter DW, Batteli LA, Wolfarth MG, Richardson DL, Ma Q (2015). Pathologic and molecular profiling of rapid-onset fibrosis and inflammation induced by multi-walled carbon nanotubes.. Arch Toxicol.

[r21] Ehrlich M, Lacey M (2013). DNA hypomethylation and hemimethylation in cancer.. Adv Exp Med Biol.

[r22] Fragou D, Fragou A, Kouidou S, Njau S, Kovatsi L (2011). Epigenetic mechanisms in metal toxicity.. Toxicol Mech Methods.

[r23] Fröhlich E, Samberger C, Kueznik T, Absenger M, Roblegg E, Zimmer A (2009). Cytotoxicity of nanoparticles independent from oxidative stress.. J Toxicol Sci..

[r24] Furukawa Y, Aizawa Y, Okada M, Watanabe M, Niitsuya M, Kotani M (2002). Negative effect of photocopier toner on alveolar macrophages determined by *in vitro* magnetometric evaluation.. Ind Health.

[r25] Gminski R, Decker K, Heinz C, Seidel A, Könczöl M, Goldenberg E (2011). Genotoxic effects of three selected black toner powders and their dimethyl sulfoxide extracts in cultured human epithelial A549 lung cells *in vitro*.. Environ Mol Mutagen.

[r26] Gong C, Tao G, Yang L, Liu J, Liu Q, Zhuang Z (2010). SiO_2_ nanoparticles induce global genomic hypomethylation in HaCaT cells.. Biochem Biophys Res Commun.

[r27] Hayden PJ, Bolmarcich J, Armento A, Jackson GR Jr, Hackett TL, Knight TL, et al (2009). Role of Toll-like receptor (TLR) activation in asthma exacerbation: experiments with in vitro models of human airway epithelial cells (EpiAirway) and epithelial cell/fibroblast co-cultures (EpiAirway-FT).. In: American Thoracic Society Annual Meeting, 15–20 May 2009. San Diego, CA, 509.

[r28] He C, Morawska L, Taplin L (2007). Particle emission characteristics of office printers.. Environ Sci Technol.

[r29] He YF, Li BZ, Li Z, Liu P, Wang Y, Tang Q (2011). Tet-mediated formation of 5-carboxylcytosine and its excision by TDG in mammalian DNA.. Science.

[r30] Hillegass JM Miller JM MacPherson MB Westbom CM Sayan M Thompson JK 2013 Asbestos and erionite prime and activate the NLRP3 inflammasome that stimulates autocrine cytokine release in human mesothelial cells. Part Fibre Toxicol 10 39; doi:10.1186/1743-8977-10-39 23937860 PMC3751315

[r31] Holder AL, Goth-Goldstein R, Lucas D, Koshland CP (2012). Particle-induced artifacts in the MTT and LDH viability assays.. Chem Res Toxicol.

[r32] IDC (International Data Corporation) (2014). IDC finds continued growth in the worldwide hardcopy peripherals market in the fourth quarter of 2013.. Press release (Framingham, MA) 19 February.

[r33] Ito S, Shen L, Dai Q, Wu SC, Collins LB, Swenberg JA (2011). Tet proteins can convert 5-methylcytosine to 5-formylcytosine and 5-carboxylcytosine.. Science.

[r34] Jin SG, Jiang Y, Qiu R, Rauch TA, Wang Y, Schackert G (2011). 5-Hydroxymethylcytosine is strongly depleted in human cancers but its levels do not correlate with *IDH1* mutations.. Cancer Res.

[r35] Jones PA (2012). Functions of DNA methylation: islands, start sites, gene bodies and beyond.. Nat Rev Genet.

[r36] Khatri M, Bello D, Gaines P, Martin J, Pal AK, Gore R (2013a). Nanoparticles from photocopiers induce oxidative stress and upper respiratory tract inflammation in healthy volunteers.. Nanotoxicology.

[r37] Khatri M Bello D Pal AK Cohen JM Woskie S Gassert T 2013b Evaluation of cytotoxic, genotoxic and inflammatory responses of nanoparticles from photocopiers in three human cell lines. Part Fibre Toxicol 10 42; doi:10.1186/1743-8977-10-42 23968360 PMC3766213

[r38] Kimura A, Miyata A, Honma M (2013). A combination of *in vitro* comet assay and micronucleus test using human lymphoblastoid TK6 cells.. Mutagenesis.

[r39] Koturbash I Scherhag A Sorrentino J Sexton K Bodnar W Tryndyak V 2011 Epigenetic alterations in liver of C57BL/6J mice after short-term inhalational exposure to 1,3-butadiene. Environ Health Perspect 119 635 640; doi:10.1289/ehp.1002910 21147608 PMC3094413

[r40] Koturbash I, Simpson NE, Beland FA, Pogribny IP (2012). Alterations in histone H4 lysine 20 methylation: implications for cancer detection and prevention.. Antioxid Redox Signal.

[r41] L’Azou B Jorly J On D Sellier E Moisan F Fleury-Feith J 2008 In vitro effects of nanoparticles on renal cells. Part Fibre Toxicol 5 22; doi:10.1186/1743-8977-5-22 19099552 PMC2621238

[r42] Lacy P, Stow JL (2011). Cytokine release from innate immune cells: association with diverse membrane trafficking pathways.. Blood.

[r43] Li Z, Cai X, Cai CL, Wang J, Zhang W, Petersen BE (2011). Deletion of *Tet2* in mice leads to dysregulated hematopoietic stem cells and subsequent development of myeloid malignancies.. Blood.

[r44] Liu JY, Hu JH, Zhu QG, Li FQ, Wang J, Sun HJ (2007). Effect of matrine on the expression of substance P receptor and inflammatory cytokines production in human skin keratinocytes and fibroblasts.. Int Immunopharmacol.

[r45] Lu X Miousse IR Pirela SV Melnyk S Koturbash I Demokritou P 2015 Short-term exposure to engineered nanomaterials affects cellular epigenome. Nanotoxicology; doi:10.3109/17435390.2015.1025115 [Online 4 May 2015] PMC4633390 25938281

[r46] Madrigano J Baccarelli A Mittleman MA Wright RO Sparrow D Vokonas PS 2011 Prolonged exposure to particulate pollution, genes associated with glutathione pathways, and DNA methylation in a cohort of older men. Environ Health Perspect 119 977 982; doi:10.1289/ehp.1002773 21385671 PMC3222977

[r47] Miousse IR, Chalbot MC, Aykin-Burns N, Wang X, Basnakian A, Kavouras IG (2014a). Epigenetic alterations induced by ambient particulate matter in mouse macrophages.. Environ Mol Mutagen.

[r48] Miousse IR, Chalbot MC, Lumen A, Ferguson A, Kavouras IG, Koturbash I (2015). Response of transposable elements to environmental stressors.. Mutat Res Rev Mutat Res.

[r49] Miousse IR, Shao L, Chang J, Feng W, Wang Y, Allen AR (2014b). Exposure to low dose ^56^Fe-ion radiation induces long-term epigenetic alterations in mouse bone marrow hematopoietic progenitor and stem cells.. Radiat Res.

[r50] Mittal S Pandey AK 2014 Cerium oxide nanoparticles induced toxicity in human lung cells: role of ROS mediated DNA damage and apoptosis. BioMed Res Int 2014 891934; doi:10.1155/2014/891934 PMC4058670 24987704

[r51] Monteiro-Riviere NA, Inman AO, Zhang LW (2009). Limitations and relative utility of screening assays to assess engineered nanoparticle toxicity in a human cell line.. Toxicol Appl Pharmacol.

[r52] Morawska L, He C, Johnson G, Jayaratne R, Salthammer T, Wang H (2009). An investigation into the characteristics and formation mechanisms of particles originating from the operation of laser printers.. Environ Sci Technol.

[r53] Morimoto Y, Oyabu T, Horie M, Kambara T, Izumi H, Kuroda E (2013). Pulmonary toxicity of printer toner following inhalation and intratracheal instillation.. Inhal Toxicol.

[r54] Nyga A, Hart A, Tetley TD (2015). Importance of the HIF pathway in cobalt nanoparticle-induced cytotoxicity and inflammation in human macrophages.. Nanotoxicology.

[r55] Pal AK, Bello D, Cohen J, Demokritou P (2015). Implications of *in vitro* dosimetry on toxicological ranking of low aspect ratio engineered nanomaterials.. Nanotoxicology.

[r56] Pirela S, Molina R, Watson C, Cohen JM, Bello D, Demokritou P (2013). Effects of copy center particles on the lungs: a toxicological characterization using a *Balb/c* mouse model.. Inhal Toxicol.

[r57] Pirela SV, Pyrgiotakis G, Bello D, Thomas T, Castranova V, Demokritou P (2014). Development and characterization of an exposure platform suitable for physico-chemical, morphological and toxicological characterization of printer-emitted particles (PEPs).. Inhal Toxicol.

[r58] Pirela SV, Sotiriou GA, Bello D, Shafer M, Bunker KL, Castranova V (2015). Consumer exposures to laser printer-emitted engineered nanoparticles: a case study of life-cycle implications from nano-enabled products.. Nanotoxicology.

[r59] Ritter M Mennerich D Weith A Seither P 2005 Characterization of Toll-like receptors in primary lung epithelial cells: strong impact of the TLR3 ligand poly(I:C) on the regulation of Toll-like receptors, adaptor proteins and inflammatory response. J Inflamm (Lond) 2 16; doi:10.1186/1476-9255-2-16 16316467 PMC1315317

[r60] Rudin CM, Thompson CB (2001). Transcriptional activation of short interspersed elements by DNA-damaging agents.. Genes Chromosomes Cancer.

[r61] Setyawati MI Tay CY Chia SL Goh SL Fang W Neo MJ 2013 Titanium dioxide nanomaterials cause endothelial cell leakiness by disrupting the homophilic interaction of VE-cadherin. Nat Commun 4 1673; doi:10.1038/ncomms2655 23575677

[r62] Sisler JD, Pirela SV, Friend S, Farcas M, Schwegler-Berry D, Shvedova A (2015). Small airway epithelial cells exposure to printer-emitted engineered nanoparticles induces cellular effects on human microvascular endothelial cells in an alveolar-capillary co-culture model.. Nanotoxicology.

[r63] Smith ZD, Chan MM, Mikkelsen TS, Gu H, Gnirke A, Regev A (2012). A unique regulatory phase of DNA methylation in the early mammalian embryo.. Nature.

[r64] Sotiriou GA, Diaz E, Long MS, Godleski J, Brain J, Pratsinis SE (2012). A novel platform for pulmonary and cardiovascular toxicological characterization of inhaled engineered nanomaterials.. Nanotoxicology.

[r65] Sriram K, Lin GX, Jefferson AM, Roberts JR, Andrews RN, Kashon ML (2012). Manganese accumulation in nail clippings as a biomarker of welding fume exposure and neurotoxicity.. Toxicology.

[r66] Tang T, Gminski R, Könczöl M, Modest C, Armbruster B, Mersch-Sundermann V (2012). Investigations on cytotoxic and genotoxic effects of laser printer emissions in human epithelial A549 lung cells using an air/liquid exposure system.. Environ Mol Mutagen.

[r67] Terasaki N Goodier JL Cheung LE Wang YJ Kajikawa M Kazazian HH Jr 2013 *In vitro* screening for compounds that enhance human L1 mobilization. PloS One 8 e74629; doi:10.1371/journal.pone.0074629 24040300 PMC3770661

[r68] Wan R, Mo Y, Feng L, Chien S, Tollerud DJ, Zhang Q (2012). DNA damage caused by metal nanoparticles: involvement of oxidative stress and activation of ATM.. Chem Res Toxicol.

[r69] Wang H, He C, Morawska L, McGarry P, Johnson G (2012). Ozone-initiated particle formation, particle aging, and precursors in a laser printer.. Environ Sci Technol.

[r70] Watson C, Ge J, Cohen J, Pyrgiotakis G, Engelward BP, Demokritou P (2014). High-throughput screening platform for engineered nanoparticle-mediated genotoxicity using CometChip technology.. ACS Nano.

[r71] Wilhelmi V Fischer U Weighardt H Schulze-Osthoff K Nickel C Stahlmecke B 2013 Zinc oxide nanoparticles induce necrosis and apoptosis in macrophages in a p47phox- and Nrf2-independent manner. PloS One 8 e65704; doi:10.1371/journal.pone.0065704 23755271 PMC3670863

[r72] Zarogiannis SG, Filippidis AS, Fernandez S, Jurkuvenaite A, Ambalavanan N, Stanishevsky A (2013). Nano-TiO_2_ particles impair adhesion of airway epithelial cells to fibronectin.. Respir Physiol Neurobiol.

[r73] Zeidler-Erdely PC, Battelli LA, Salmen-Muniz R, Li Z, Erdely A, Kashon ML (2011). Lung tumor production and tissue metal distribution after exposure to manual metal ARC-stainless steel welding fume in A/J and C57BL/6J mice.. J Toxicol Environ Health A.

