# Supplementary material for: Effects of Laser Printer–Emitted Engineered Nanoparticles on Cytotoxicity, Chemokine Expression, Reactive Oxygen Species, DNA Methylation, and DNA Damage: A Comprehensive in Vitro Analysis in Human Small Airway Epithelial Cells, Macrophages, and Lymphoblasts
Source: Environ Health Perspect. 2015 Jun 16;124(2):210–9. doi: 10.1289/ehp.1409582 (PMC4749083; doi:10.1289/ehp.1409582)
Supplement: (826 KB) PDF [file ehp.1409582.s001.acco.pdf]

Environ Health Perspect
DOI: 10.1289/ehp.1409582

Note to Readers: *EHP* strives to ensure that all journal content is accessible to all readers. However, some figures and Supplemental Material published in *EHP* articles may not conform to 508 standards due to the complexity of the information being presented. If you need assistance accessing journal content, please contact ehp508@niehs.nih.gov. Our staff will work with you to assess and meet your accessibility needs within 3 working days.

Supplemental Material

Effects of Laser Printer–Emitted Engineered Nanoparticles on Cytotoxicity, Chemokine Expression, Reactive Oxygen Species, DNA Methylation, and DNA Damage: A Comprehensive *in Vitro* Analysis in Human Small Airway Epithelial Cells, Macrophages, and Lymphoblasts

Sandra V. Pirela, Isabelle R. Miousse, Xiaoyan Lu, Vincent Castranova, Treye Thomas, Yong Qian, Dhimiter Bello, Lester Kobzik, Igor Koturbash, and Philip Demokritou

Table of Contents

Supplemental Material, Part A

Dosimetric considerations for *in vitro* testing – Example of calculations

Table S1. Summary of parameters used in the *in vivo* lung Multiple Path Particle Deposition model (MPPD2).

Table S2. Assays for determination of LINE-1 and *Alu* methylation.

Table S3. Assays for determination of gene expression.

Table S4. *In vitro* administered and delivered doses of SiO_2 and MS-WF.

Figure S1. Hydrodynamic diameter as a function of DSE for PEPs ($PM_{0.1}$) and MS-WF. DSE_{cr}: critical delivered sonication energy, energy required for minimal agglomeration.

Figure S2. Fraction of administered dose deposited, f_D, as a function of *in vitro* exposure time for PEPs ($PM_{0.1}$), SiO_2 and MS-WF calculated using the agglomeration diameter and estimated effective density. Plots are presented for the tested materials in the two media formulations (RPMI/10% FBS and SAGM).

Figure S3. Deposition mass flux (left axis) and deposition fraction (right axis) as a function of airway generation number.

Figure S4. Quantitative DNA damage assessment (Comet assay) of human lymphoblasts (TK6 cells) exposed for 4 hours to PEPs at various doses. All values are represented as mean ± SE.

References for Supplemental Material, Part A.

Supplemental Material, Part A

Dosimetric considerations for *in vitro* testing – Example of calculations

The following example shows the step-by-step calculations performed to arrive at the number of hours of PEPs inhalation that match the delivered-to-cell doses (*e.g.*, 0.5 μg/ml) used for the two cell lines (SAECs, THP-1) in the study.

1. **Choose the administered dose of interest used in the experiment to determine the corresponding inhalation exposure to PEPs.**
   For this example, we chose the administered dose of 0.5 μg/ml.

2. **Calculate the mass administered (μg) per well in a 96-well plate**
   Mass administered-to-cell in one well (μg) = administered dose * administered volume
   Mass administered-to-cell in one well (μg) = (0.5 μg/ml) * (0.1 ml) = 0.05 μg

3. **Converting the administered mass to delivered to cell dose as a function of the *in vitro* exposure time (t = 24 hrs) using the *in vitro* dosimetric methodology (Cohen et al. 2014):**

   The fraction of administered particle mass that is deposited on the cells in a standard 96-well plate as a function of *in vitro* exposure time (f_D) is calculated. For a 24-hour *in vitro* exposure, the f_D was found to be 1.0 for particles suspended in SAGM (SAECs) and 0.518 for particles suspended in RPMI/10%FBS (THP-1). Therefore, the delivered to cell *in vitro* mass is as follows:

   SAECs
   Delivered to cell mass (μg) = f_D*administered to cell mass (μg)=(1.00 * 0.05 μg) = 0.05 μg

   THP-1
   Delivered to cell mass (μg) = f_D*administered to cell mass (μg)=(0.518 * 0.05 μg) = 0.0259 μg

4. **Calculate the mass delivered-to-cells per well surface area (μg/m^2).**
   Dose delivered-to-cells per area (μg/m^2) = Mass delivered-to-cells (μg) / Surface area of one well in a 96-well plate (m^2)

   SAECs
   Dose delivered-to-cells per well area (μg/m^2) = (0.05 μg) / (0.000032 m^2) = 1,562.5 μg/m^2

   THP-1
   Dose delivered-to-cells per well area (μg/m^2) = (0.0259 μg) / (0.000032 m^2) = 809.4 μg/m^2

5. **Obtain deposited mass flux from MPPD2 model using the airborne nanoparticle size distribution values (*i.e.*, count median diameter, geometric standard deviation, particle mass concentration) and the human breathing parameters of a resting individual (*i.e.*, tidal volume, breathing frequency, inspiratory fraction, pause fraction, functional residual capacity, head volume, breathing route). These values can be found on Table 2.**

$$\textit{deposition mass flux} = 1.732\ \mu g/m^2 \bullet min$$

6. **Calculate the human inhalation PEPs exposure duration (min) that matches the previously calculated *in vitro* dose delivered to cells by area (μg/m^2).**

SAECs

$T_{inhalation\ exposure}$ (min) = ?
Deposition mass flux (μg/m^2•min) = 1.732 μg/m^2•min
Dose delivered-to-cells by area (μg/m^2) = 1,562.5 μg/m^2

$T_{inhalation\ exposure}$ (min) = Mass delivered-to-cells per area (μg/m^2) / Deposition mass flux (μg/m^2•min)

$T_{inhalation\ exposure}$ = 902.14 min = 15.04 hours

THP-1

$T_{inhalation\ exposure}$ (min) = ?
Deposition mass flux (μg/m^2•min) = 1.732 μg/m^2•min
Dose delivered-to-cells by area (μg/m^2) = 809.4 μg/m^2

$T_{inhalation\ exposure}$ (min) = Mass delivered-to-cells per area (μg/m^2) / Deposition mass flux (μg/m^2•min)

$T_{inhalation\ exposure}$ = 467.32 min = 7.78 hours

Table S1. Summary of parameters used in the *in vivo* lung Multiple Path Particle Deposition model (MPPD2).

Human Model	Breathing Parameters	Airborne Nanoparticle Distribution
Functional Residual Capacity: 3300 mL	*Tidal Volume*: 625 ml	*Count Mean Diameter:* 57.45 nm
Head Volume: 50 mL	*Breathing Frequency:* 12 breaths/ min	*Geometric Standard Deviation*: 1.67
Breathing Route: Nasal	*Inspiratory Fraction*: 0.5	*Mass Concentration*: 23.86 μg/m^3
	Pause Fraction: 0.0	

Table S2. Assays for determination of LINE-1 and *Alu* methylation.

	**Forward Primer**	**Reverse Primer**
**Methylation**		
LINE1 5'UTR (L1P1)	AAAGAAAGGGGTGACGGACG	TACCTAAGCAAGCCTGGGCAA
LINE1 ORF2	TGGAACCCTTGTGCACTGTT	CCAGAAGTGGAATTGCTGGA
Alu	GCCTGTAATCCCAGCACTTT	TCTCCTGCCTCAGCCTCC
**Expression**		
LINE1 ORF2	AAATGGTGCTGGGAAAACTG	GCCATTGCTTTTGGTGTTTT
Alu	CATGGTGAAACCCCGTCTCTA	GCCTCAGCCTCCCGAGTAG

Table S3. Assays for determination of gene expression.

Gene Symbol	Assay Name	RefSeq #	Source
DNMT1	Hs.PT.56.28037916	NM_001130823	Integrated DNA Technologies
DNMT3A	Hs01027166_m1	NM_022552.4	Life Technologies
DNMT3B	Hs00171876_m1	NM_001207055.1	Life Technologies
GAPDH	Hs.PT.56.589810.g	NM_001256799	Integrated DNA Technologies
HMOX1	Hs01110250_m1	NM_002133.2	Life Technologies
TET1	Hs00286756_m1	NM_030625.2	Life Technologies
TET2	Hs00325999_m1	NM_001127208.2	Life Technologies
TET3	Hs00379125_m1	NM_144993.1	Life Technologies
UHRF1	Hs01086727_m1	NM_001048201.1	Life Technologies

Table S4. *In vitro* administered and delivered doses of SiO_2 and MS-WF.

Particle	Cell administered dose[a] (µg/mL)	Cell delivered dose[a] (µg/mL) SAEC	Cell delivered dose[a] (µg/mL) THP-1
SiO_2	0.5	0.5	0.177
	5	5	1.77
	10	10	3.54
	20	20	7.08
	30	30	10.62
	40	40	14.16
	100	100	35.4
Mild steel welding fumes (MS-WF)	0.5	0.5	0.5
	5	5	5
	10	10	10
	20	20	20
	30	30	30
	40	40	40
	100	100	100

Notes:
[a] *In vitro* administered- and delivered doses are based on a 24-hour *in vitro* exposure.

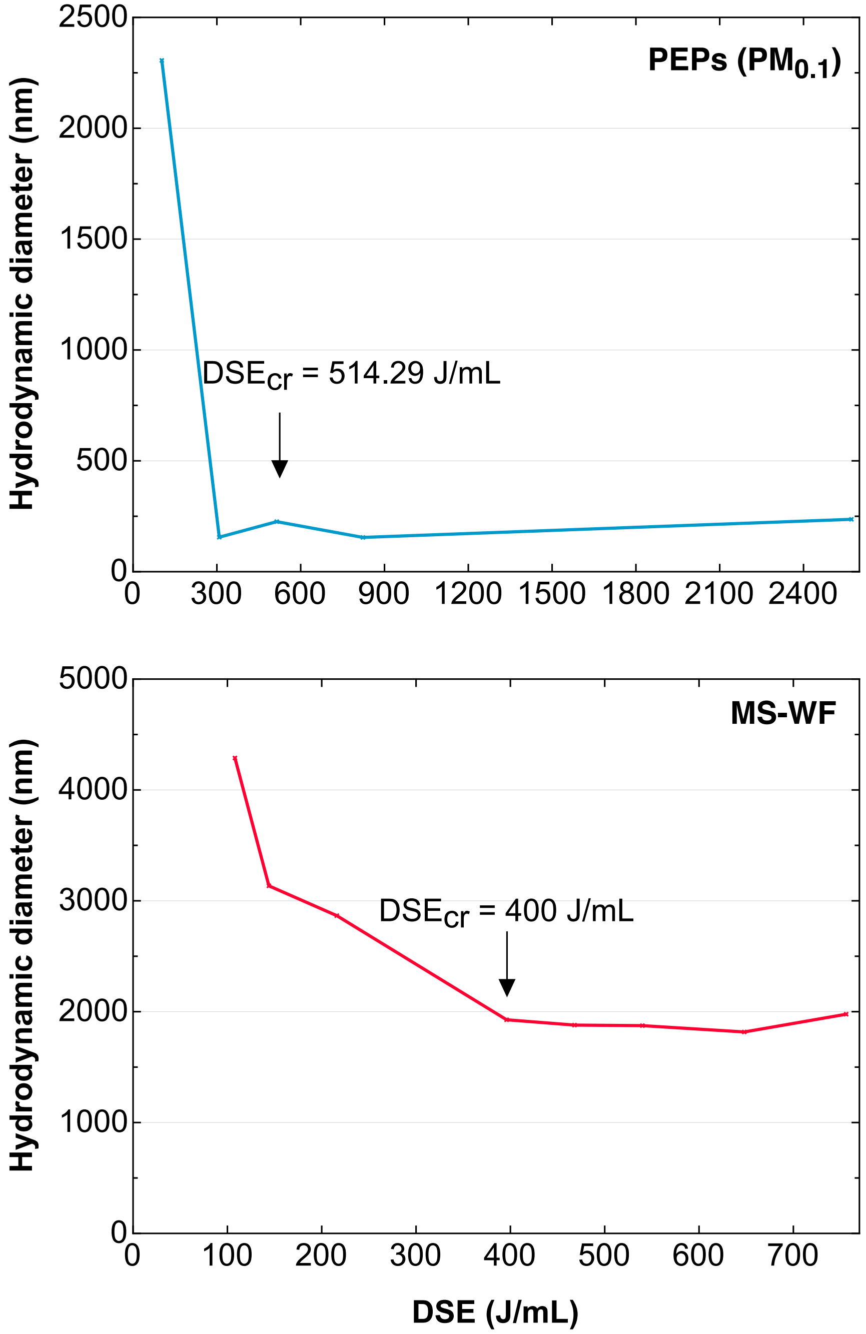

Figure S1. Hydrodynamic diameter as a function of DSE for PEPs ($PM_{0.1}$) and MS-WF. DSE_{cr}: critical delivered sonication energy, energy required for minimal agglomeration.

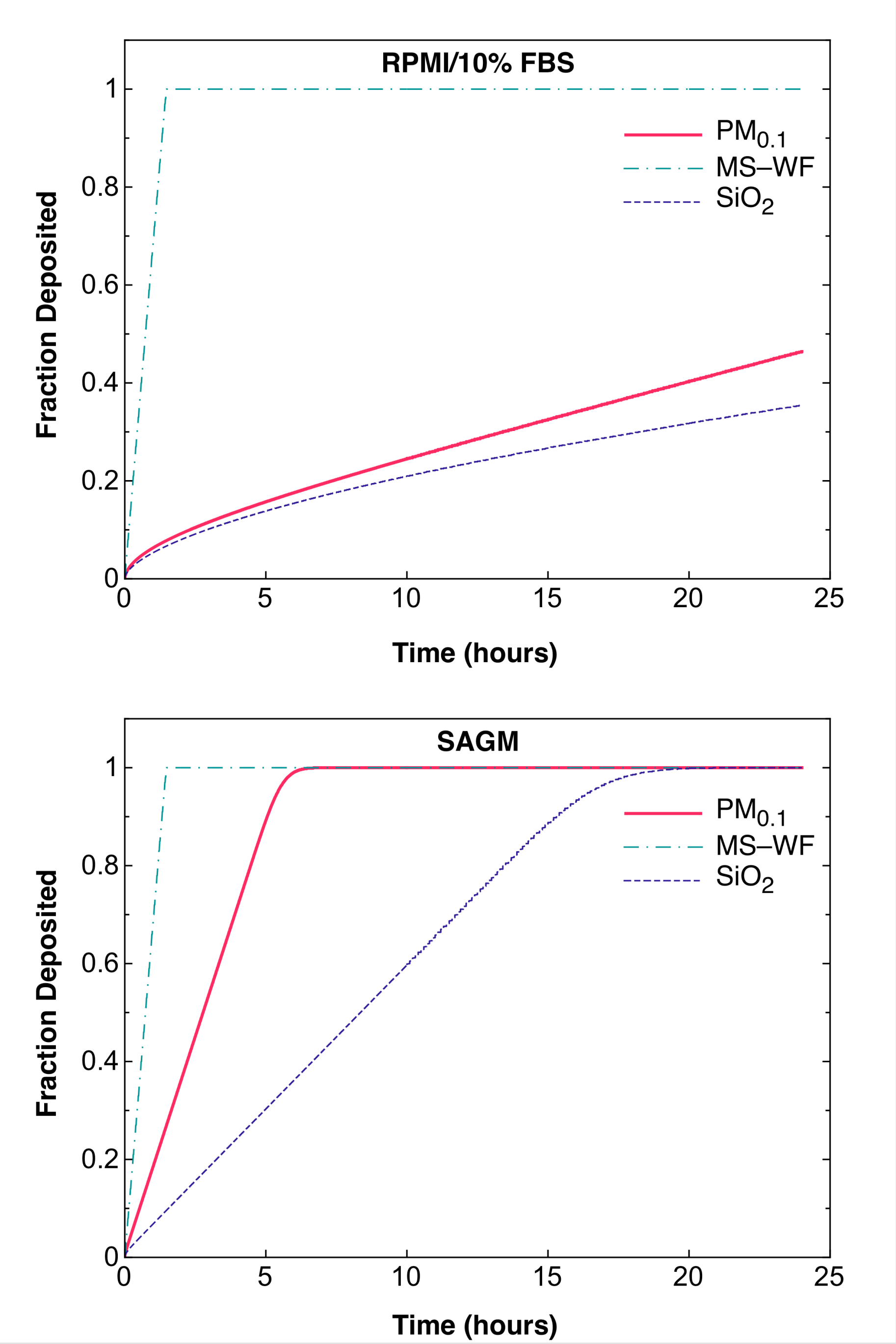

Figure S2. Fraction of administered dose deposited, f_D, as a function of *in vitro* exposure time for PEPs ($PM_{0.1}$), SiO_2 and MS-WF calculated using the agglomeration diameter and estimated effective density. Plots are presented for the tested materials in the two media formulations (RPMI/10% FBS and SAGM).

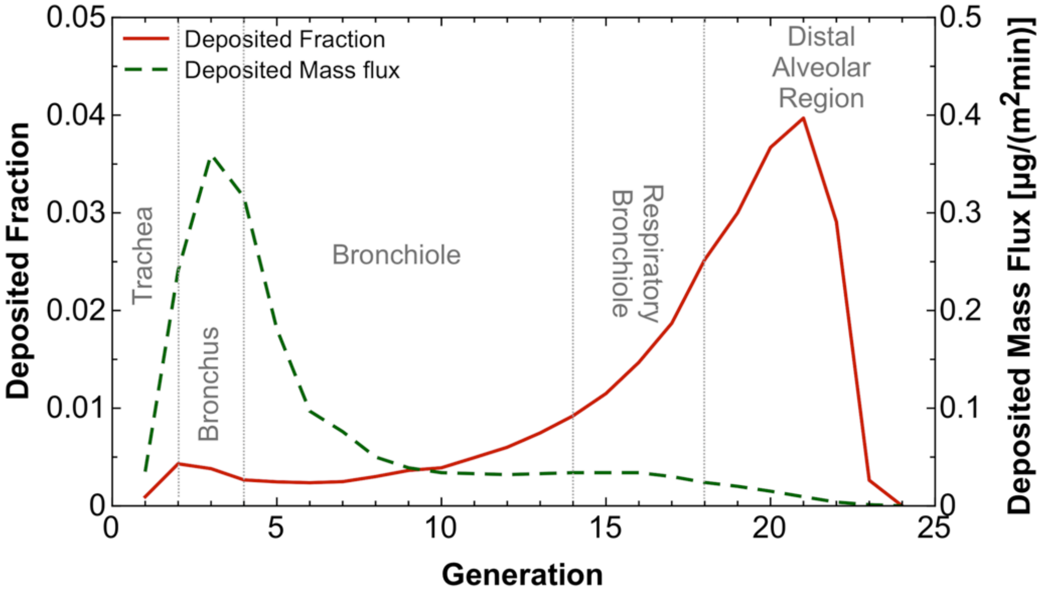

Figure S3. Deposition mass flux (left axis) and deposition fraction (right axis) as a function of airway generation number.

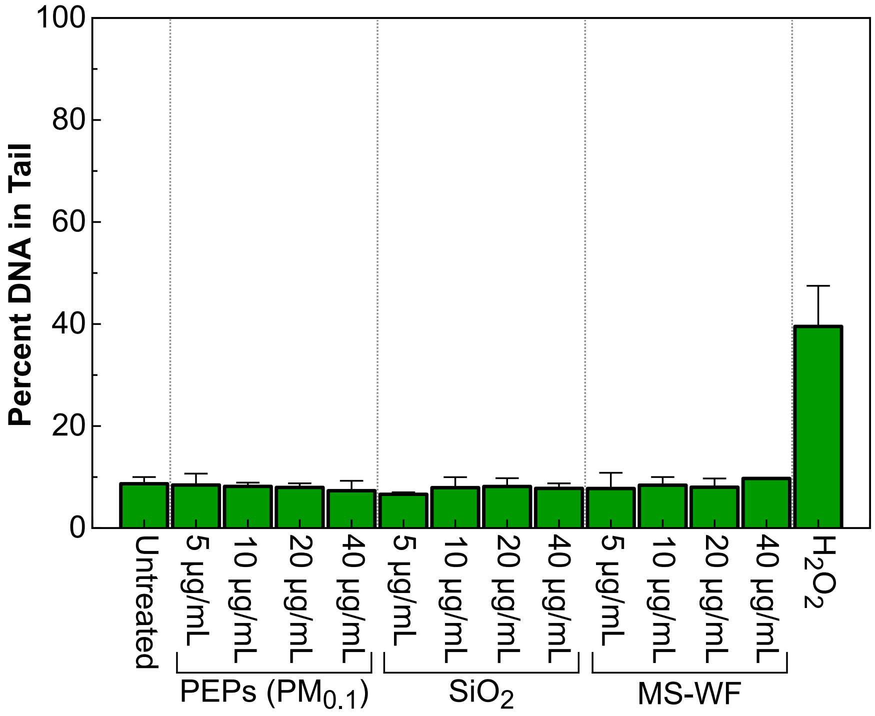

Figure S4. Quantitative DNA damage assessment (Comet assay) of human lymphoblasts (TK6 cells) exposed for 4 hours to PEPs at various doses. All values are represented as mean ± SE.

References for Supplemental Material, Part A.

Cohen J, Deloid G, Pyrgiotakis G, Demokritou P. 2012. Interactions of engineered nanomaterials in physiological media and implications for in vitro dosimetry. Nanotoxicology. 8 Suppl 1:216-225.

Cohen JM, Teeguarden JG, Demokritou P. 2014. An integrated approach for the in vitro dosimetry of engineered nanomaterials. Part Fibre Toxicol 11:20.

DeLoid G, Cohen JM, Darrah T, Derk R, Rojanasakul L, Pyrgiotakis G, et al. 2014. Estimating the effective density of engineered nanomaterials for in vitro dosimetry. Nature communications 5:3514.
